# Supplementary material for: Demonstration of ultra-high recyclable energy densities in domain-engineered ferroelectric films
Source: Nat Commun. 2017 Dec 8;8:1999. doi: 10.1038/s41467-017-02040-y (PMC5722920; doi:10.1038/s41467-017-02040-y)
Supplement: Supplementary file 1 — Supplementary Information [file 41467_2017_2040_MOESM1_ESM.pdf]

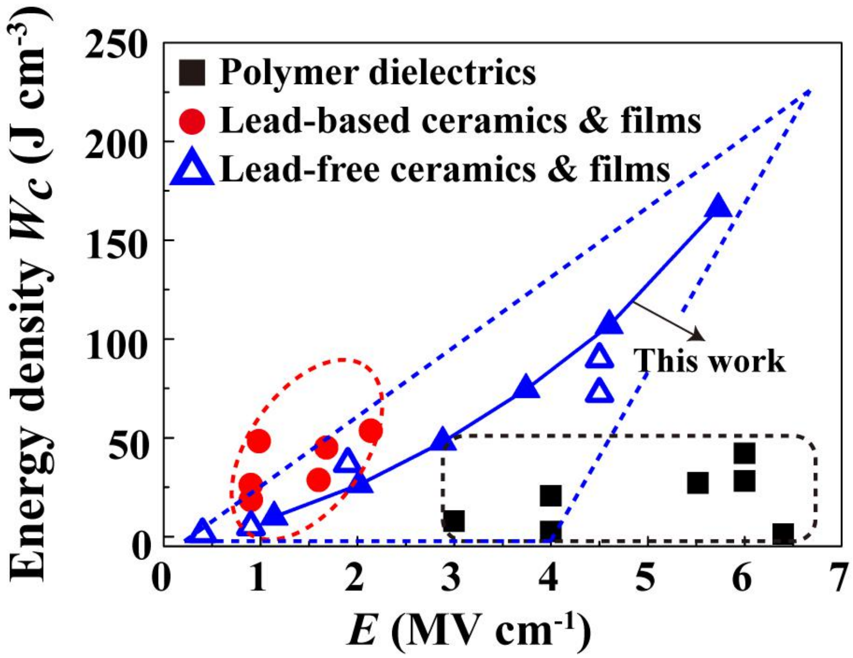


**Supplementary Figure 1: Energy densities vs. the applied electric field E.** This figure compares the (capacitive) energy densities achieved in this work (solid triangles) with those reported for other leading dielectrics.[1-17]

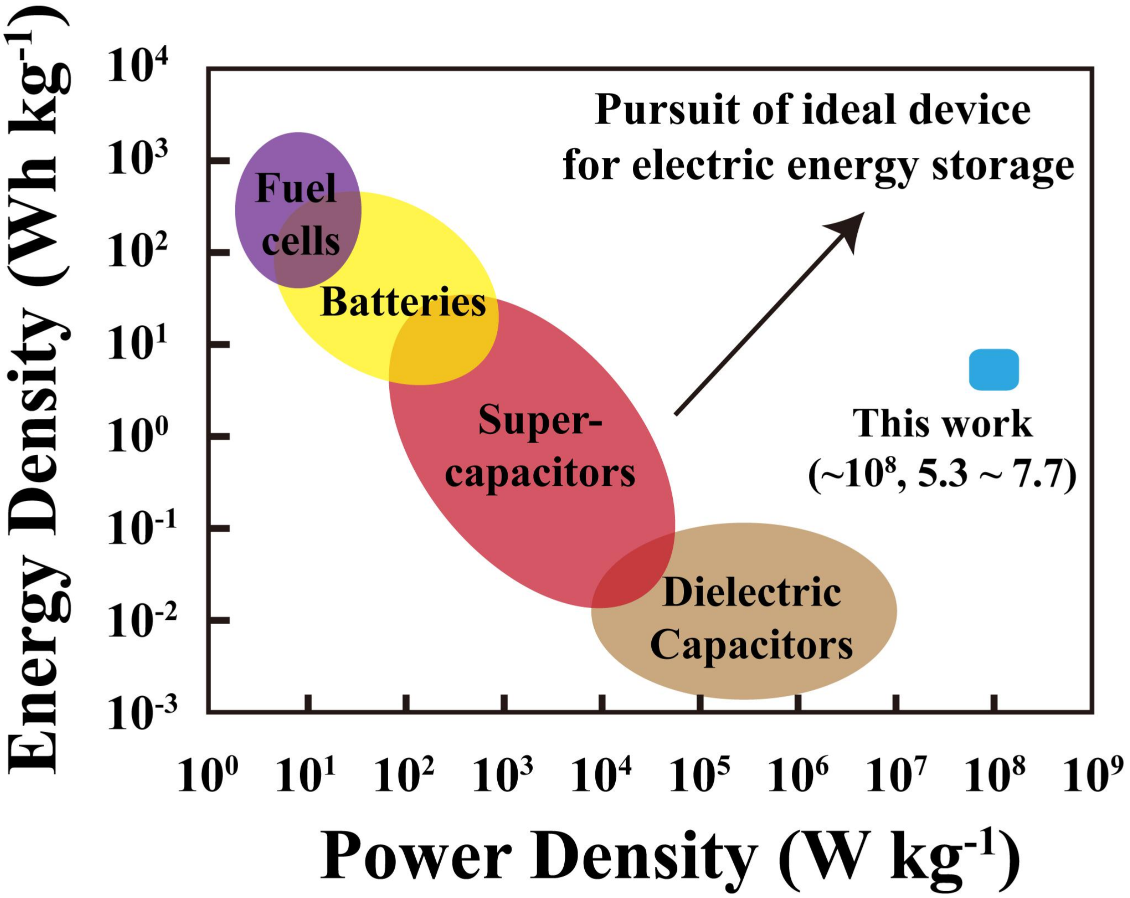


**Supplementary Figure 2: Energy and power densities of typical electrical devices for energy storage.** (R=100kΩ and C~4.5nF in the discharge experiments for estimation of the power density of the BZT thin film capacitors).

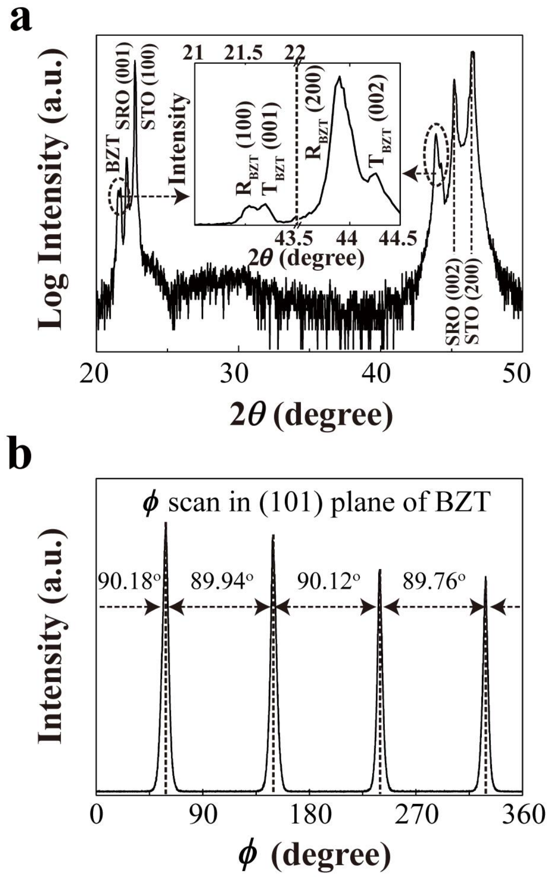


**Supplementary Figure 3: X-ray diffraction patterns.** (**a**) *2θ*-scan pattern (inset: zoom-ins around the BZT peaks) and (**b**) *Φ*-scan pattern of the (101) plane of the rhombohedral phase of a BZT(1.8 μm thick) /SRO/(100) STO thin film heterostructure.

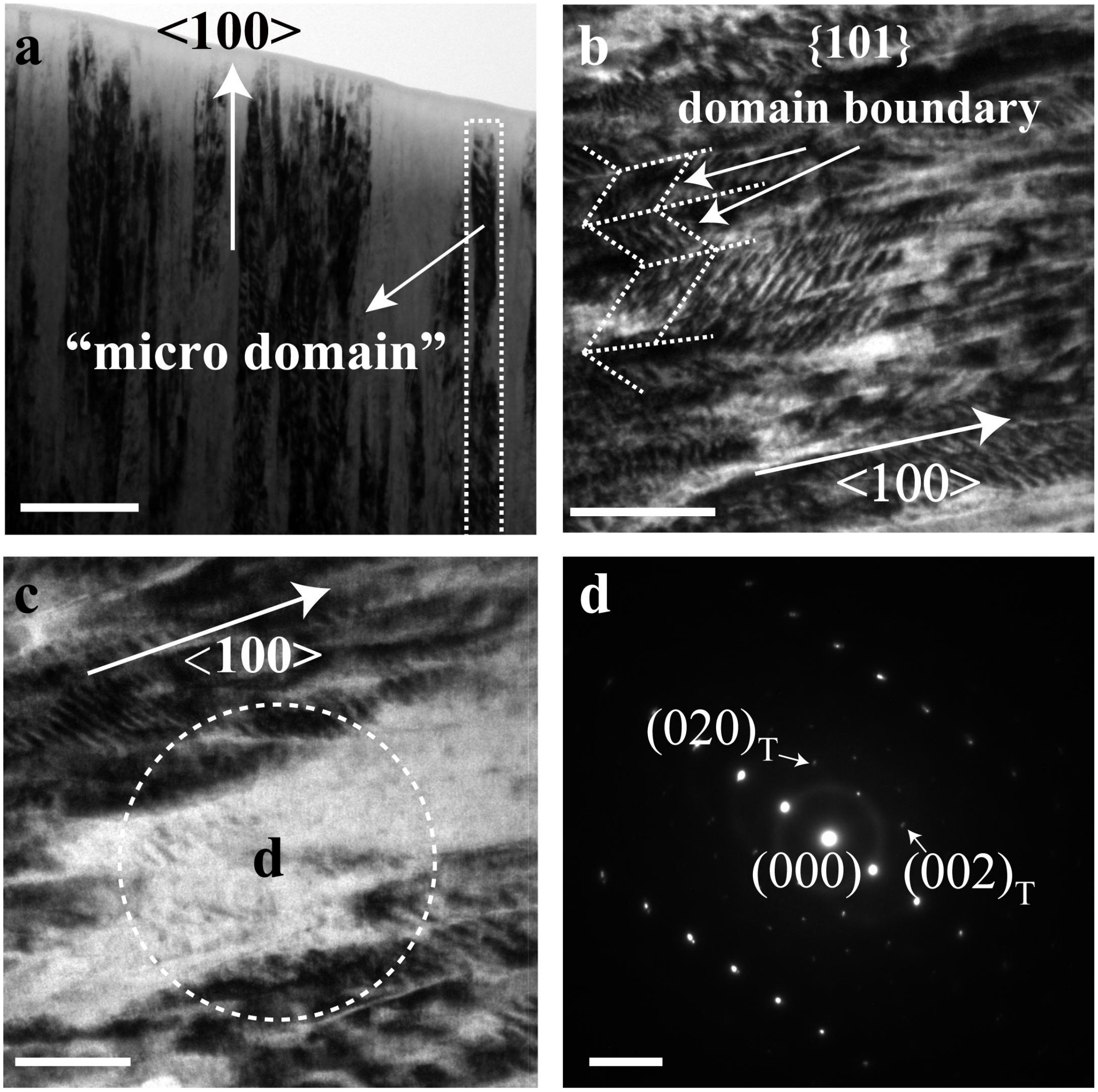


**Supplementary Figure 4: Transmission electron microscopy (TEM) of the BZT films.** (**a**) A cross-sectional bright field TEM image of a 1.8 μm thick BZT film, showing the vertically grown rhombohedral {100} "micro-domains" (Scale bar: 200 nm); (**b**) and (**c**) high magnification TEM images of a 0.7 μm thick BZT film, zig-zag shaped {101} boundaries between the nano-domains residing in the {100} "micro-domains" are marked in (**b**) (Scale bar: 50 nm), while an embedded *T*-phase domain (white) in the matrix of hierarchical rhombohedral polytwins is displayed in (**c**) (Scale bar: 50 nm); (**d**) the SAED pattern of the area centered in the white region in (**c**) with a radius of ~ 50nm, the diffraction pattern verifies existence of the embedded *T*-phase (Scale bar: 5 $nm^{-1}$).

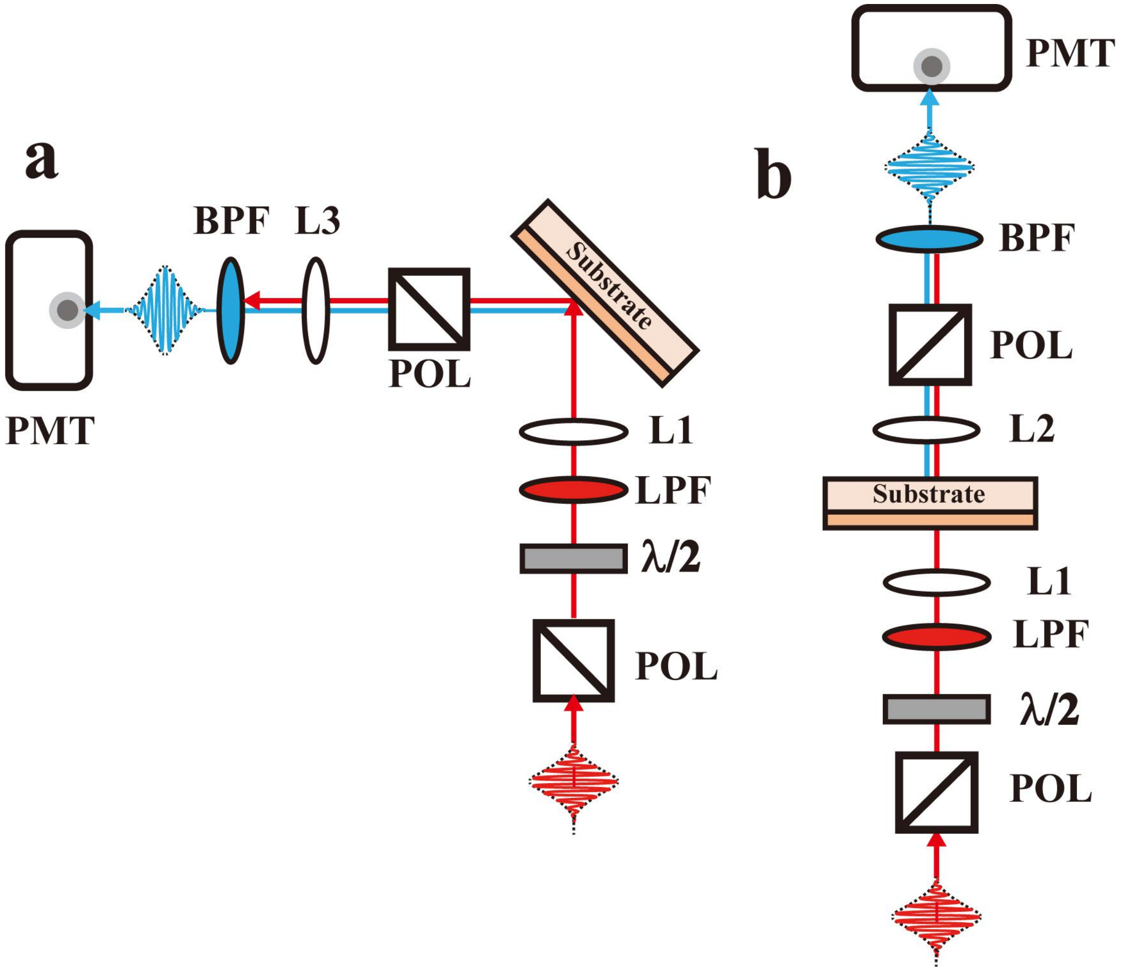
a
BPF
L3
Substrate
POL
PMT
L1
LPF
λ/2
POL
b
PMT
BPF
POL
L2
Substrate
L1
LPF
λ/2
POL

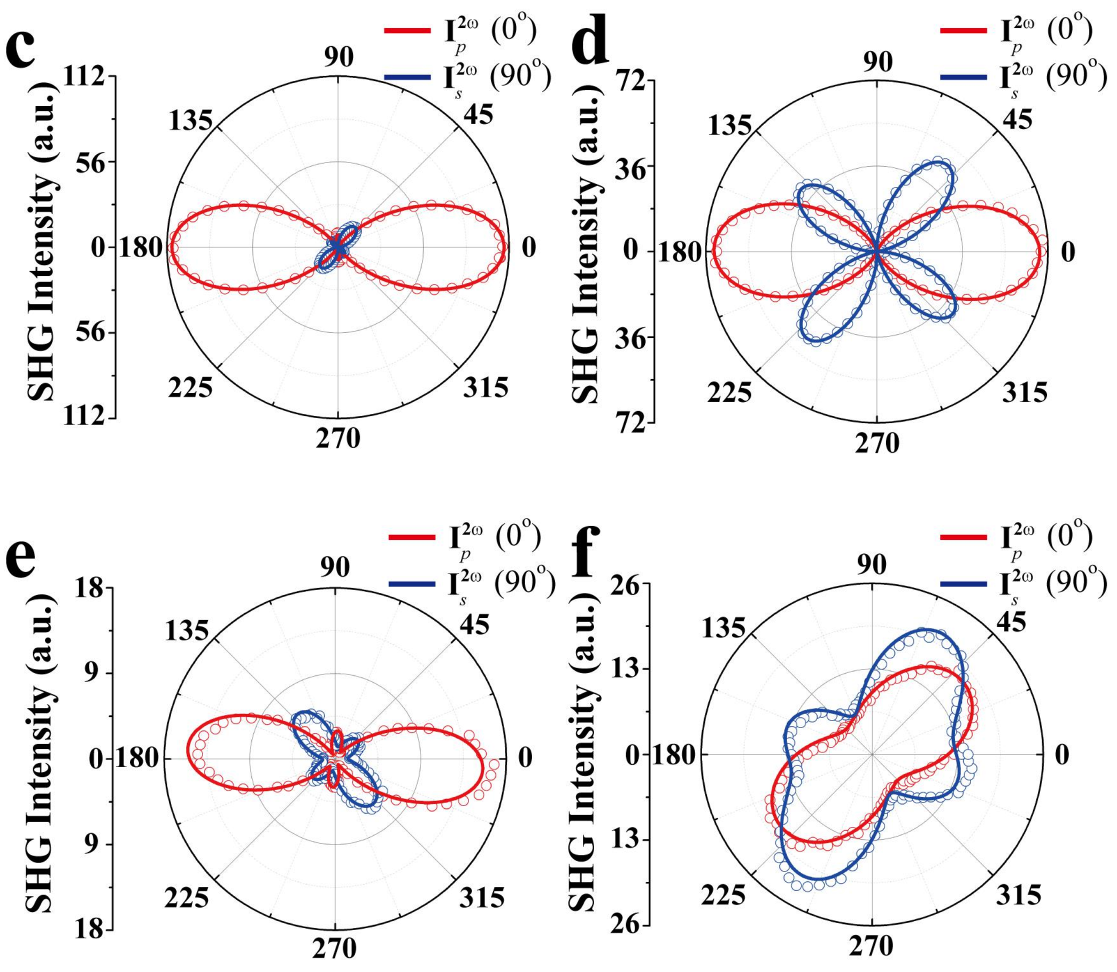


**Supplementary Figure 5: Optical second harmonic generation (SHG) measurements.** A thin film sample is tilted by $\theta = 45^{o}$ with respect to the incident light in the reflection geometry (**a**) whereas it is tilted by $\theta = 0^{o}$ with respect to the incident light in the transmission geometry (**b**); *p*-polarized SHG intensity profiles collected from (**c**) a tetragonal phase dominant region and (**d**) a rhombohedral phase dominant region of a 1.8 μm thick BZT film (on LAO) in the reflection geometry; *s*-polarized SHG intensity profiles collected from (**e**) a 0.35 μm thick BZT film (on LAO) and (**f**) the 1.8 μm thick BZT film (the same film as in **c**, **d**) in the transmission geometry.

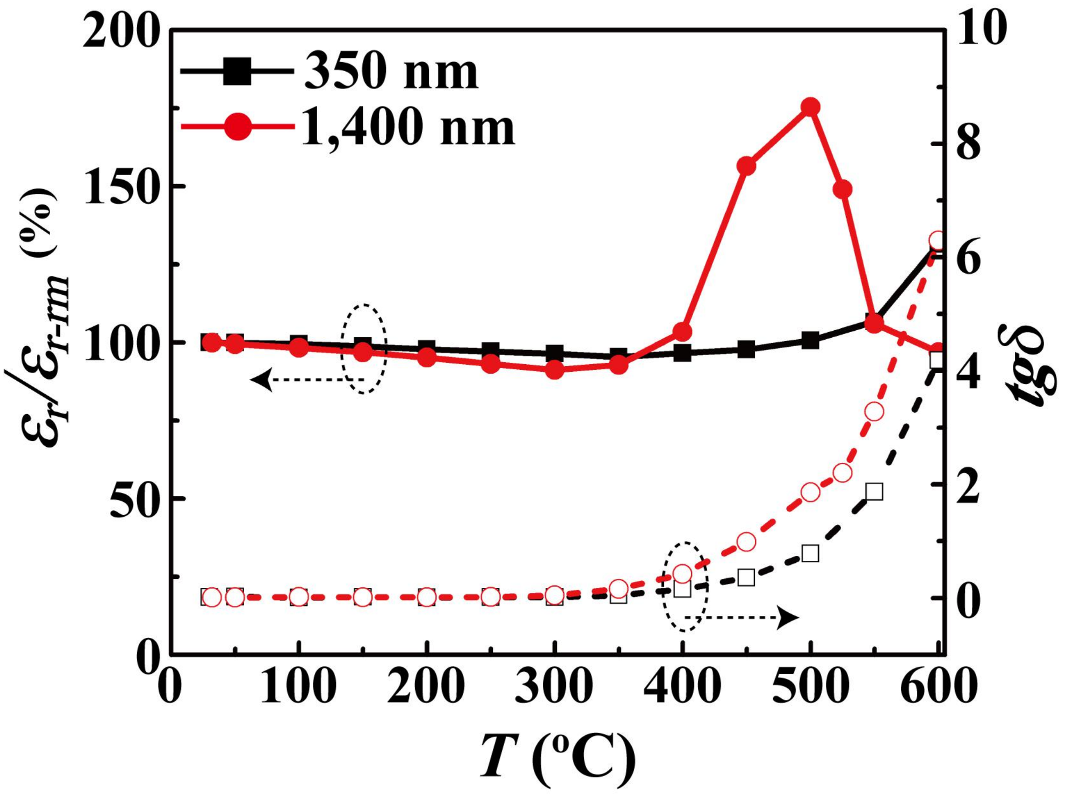


**Supplementary Figure 6: $\varepsilon_r$ -*T* and *tg*$\delta$-*T* curves.** Measured at 1 kHz for thin and thick BZT films grown on LAO ($\varepsilon_r$ were normalized to those measured at room temperature, $\varepsilon_{r\text{-}rm}$).

Note: The upper limit of the temperature controllable in the probe station is 600 °C. Due to a higher compressive misfit, the thin BZT film (350nm) showed a Curie temperature higher than 600 °C.

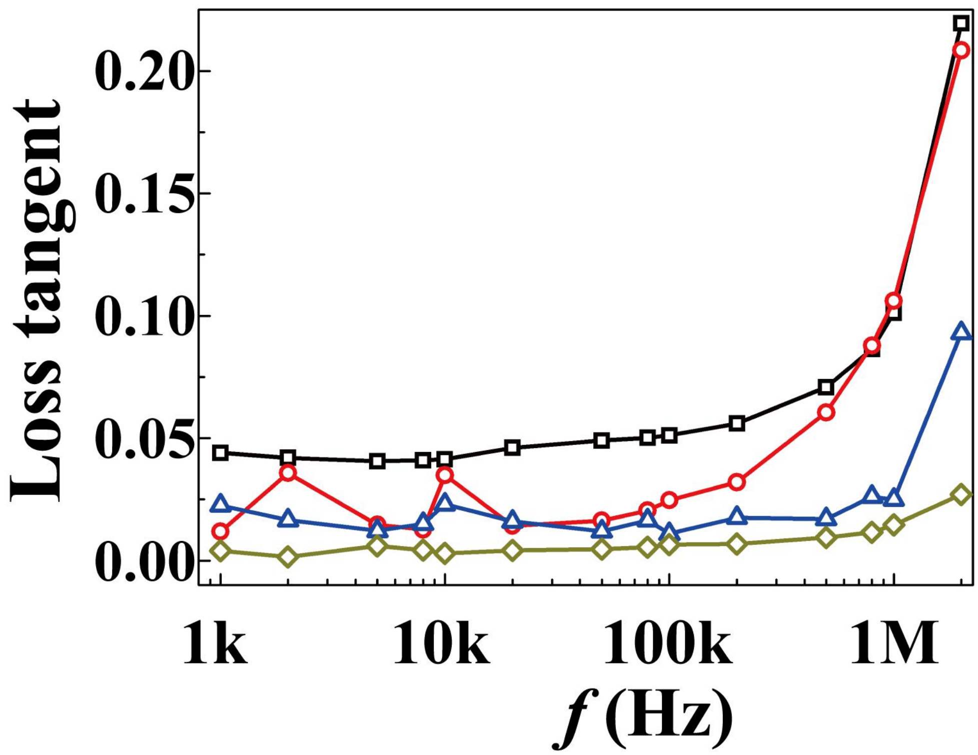


**Supplementary Figure 7: Frequency-dependent dielectric losses.** Replotted from Fig. 4d for the BZT films with different thicknesses grown on the same substrates (LAO).

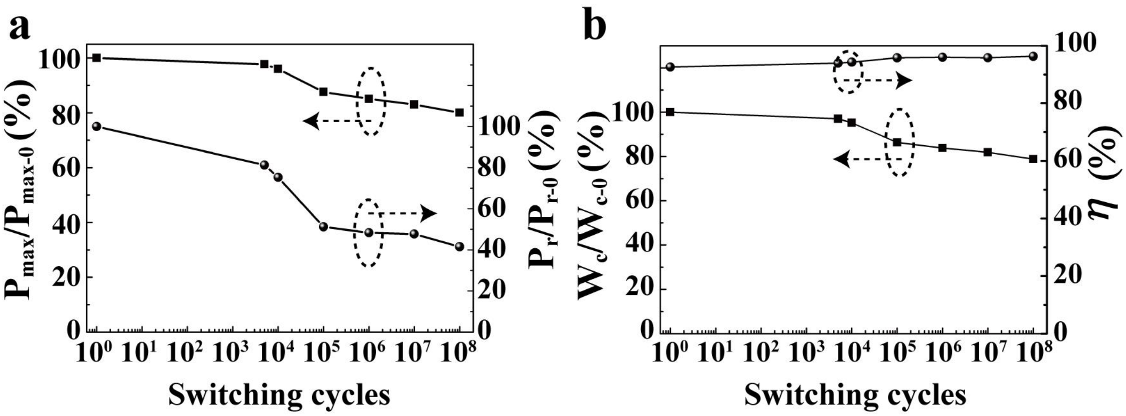


**Supplementary Figure 8: Polarizations and energy storage characteristics as functions of a cycling electric field.** (**a**) Normalized maximum and remnant polarizations $P_{max}$, $P_r$ , and (**b**) energy storage density/efficiency ($W_c$, $\eta$) of a 1.4 μm BZT film (on LAO) as functions of cycling times of a 5 kHz, 190V (1.35MV $cm^{-1}$) triangular voltage wave. **P**-**E** loops before and after cycling were measured by using a similar triangular wave (1kHz, 190V).

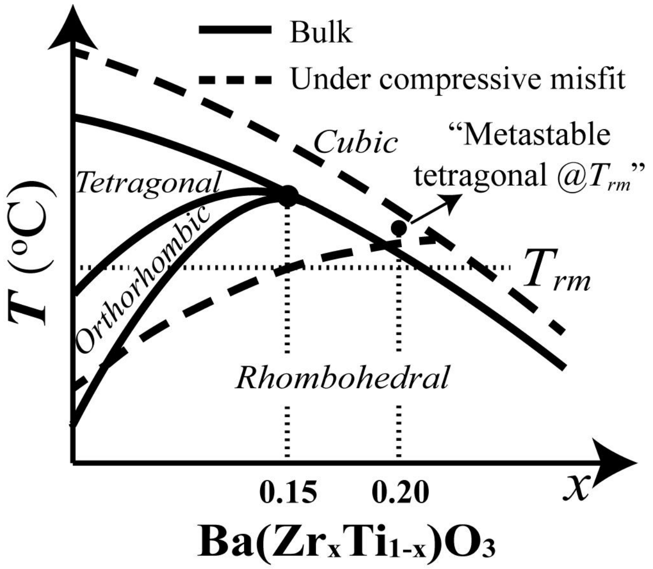


**Supplementary Figure 9: Illustrative temperature-composition phase diagram** of the $Ba(Zr_xTi_{1-x})O_3$ ferroelectric solid solution (partially shown). $T_{rm}$ : room temperature.

**Supplementary Table 1: Energy storage performances of different high energy density dielectrics.**

| Reference No. | Material | $\varepsilon_r$ | Maximum $E$ field (MV cm$^{-1}$) | Energy Density $W_C$ (J cm$^{-3}$) | Efficiency $\eta$ | Max. Operating Temperature ($^o$C) |
|---|---|---|---|---|---|---|
| 1-3 | P(VDF-CTFE) (FE) | ~47.5 | ~6 | 28-42 | ~60% -70% | 100 |
| 3 | Ternary Polymer Nanocomposite (FE) (P(VDF-CTFE)/BNNS/BT) | 12 | 5.52 | 27 | 78% | 100 |
| 4 | BOPP(commercial capacitors) (LD) | ~2.2 | 6.4 | 1.2 | --- | 105 |
| 5 | Ferroelectric polymer networks (FE) | 9.8 | 4 | 20.5 | 83% | 100 |
| 6 | c-BCB/BNNS nanocomposites (LD) | 3.1 | 4 | 2.5 | 90% | 250 |
| 7 | Neat 2-Cyanoethyltrime thoxysilane (LD) | 20 | 3 | 7.69 | 91% | --- |
| 8 | $(Pb_{0.92}La_{0.08})$-$(Zr_{0.52}Ti_{0.48})O_3$ (FE) | 1100 | 1.6 | 28.6 | ~72% | 150 |
| 9 | $(Pb_{0.91}La_{0.09})$-$(Zr_{0.65}Ti_{0.35})O_3$ (FE) | ~1400 | 1.68 | 45 | 68.4% | 200 |
| 10 | $Pb_{(1-3x/2)}La_xZr_{0.85}Ti_{0.15}O_3$ (AFE) | 520 | 2.14 | 53.5 | ~70% | 150 |
| 11 | Sr-Doped $PbZrO_3$ (AFE) | 120 | 0.9 | 18.6 | 78% | 210 |
| 12 | Eu doped $PbZrO_3$ (AFE) | ~230 | 0.9 | 26.1 | 72% | 200 |
| 13 | (042)-preferred $Pb_{0.97}La_{0.02}(Zr_{0.98}Ti_{0.02})O_3$ (AFE) | 420 | 0.984 | 48.2 | 52.3% | 120 |
| 14 | $(1-x)BaTiO_3$-$xBi(Mg,Ti)O_3$ | 900 | 1.9 | 37 | --- | 200 |
| 15 | $BaTiO_3$ Multilayers (FE) | 1800 | 0.4-0.9 | 1.4-5 | --- | --- |
| 16 | $(BCT/BZT)_n$ multilayers (FE) | 200 | 4.5 | 72.5 | 72.3% | 140 |
| 17 | $Hf_xZr_{1-x}O_2$ (AFE) | 35-40 | 4.5 | 90 | ~50% | 175 |
| This work | Engineered Poly-domain BZT films (FE) | 200-400 (350 nm-1800nm) | 2.2-**5.7** | 40-**166** | ~70%-**96%** | **300** (tgδ<5%) |

**Note:** FE: ferroelectric, AFE: antiferroelectric, LD: linear dielectric.

Dielectric permittivities are the ones measured at the remnant state or under a small field.

**Supplementary Note 1: Energy storage characteristics of a dielectric**

Densities of electrical energy stored in or released from a dielectric can be calculated from its characteristic *P*-*E* curve* by the formula:

$$W = \int EdP \approx \frac{1}{2}\varepsilon_{eff}E^2 = \frac{1}{2}\varepsilon_0\varepsilon_r^{eff}E^2 \quad (1)$$

Where $W$ is the energy density, $E$ is the applied electric field, $P$ is the polarization, $\varepsilon_{\mathrm{eff}}$ and $\varepsilon_r^{eff}$ are the effective dielectric constant and relative effective dielectric constant by the linear approximation, respectively. If $W_{\mathrm{C}}$ is used to denote the charged/stored energy density (Supplementary Equation 2), and $W_{\mathrm{re}}$ the discharged/released energy density, i.e., the recyclable energy density (Supplementary Equation 3), the energy efficiency ($\eta$) can be defined and calculated by (Supplementary Equation 4):

$$W_C = \int_0^{P_s} EdP \quad (2)$$

$$W_{re} = \int_{P_r}^{P_s} EdP \quad (3)$$

$$\eta = \frac{W_{re}}{W_c} = 1 - \frac{W_{Loss}}{W_c} \quad (4)$$

where $P_{\mathrm{s}}$ and $P_{\mathrm{r}}$ are the saturated polarization and remnant polarization of the dielectric, respectively. It is clear that for an ideal linear dielectric, $W_c = W_{re}$, and $\eta = 1$. For typical ferroelectrics (**P**-**E** curves shown in **Fig. 1(a)** and **(b)**), their energy storage characteristics ($W_{\mathrm{C}}$ and $\eta$) strongly depend on the shape of the **P**-**E** curve. Furthermore, since the frequency of a measuring electric field **E** strongly affects the shape of a **P**-**E** curve (The faster **E** is applied, the more possible a **P**-**E** curve can be extended to a higher field, due to a reduced possibility of electrical break-down. This leads to higher $W_{\mathrm{C}}$ and $\eta$ values.),

we strongly recommend to use a standard pseudo-static measuring frequency $f$ for collecting and reporting the $W_C$ and $\eta$ data. For films of a few hundred nm thick or thinner, $f$ = 1 kHz is commonly used and herein recommended. For films with sub-micron or μm level thickness, $f$ = 100 Hz is recommended.

* A ferroelectric's electric polarization (**P**) is close to its electric displacement (**D**) ( $D=\varepsilon_0 E+P=\varepsilon_0(1+\chi)E+P_0\approx\varepsilon_0\chi E+P_0=P$ , since $\chi >> 1$ in ferroelectrics), and the two notations are often interchangeably used for ferroelectrics. Rigorously, what is measured in the so-called polarization-voltage test (*P-V* test) of a ferroelectric is its *D-V* curve. The notation of polarization is used throughout this work for a better description of the domain structure, while the energy densities and efficiencies calculated from the "*P-V*" curves are correct.

**Supplementary Note 2: Energy storage performance of ferroelectrics and related materials.**

To evaluate the energy storage performance of a dielectric material, there are several key parameters to be taken into consideration (see Supplementary Table 1 below), including the stored energy density $W_C$ and energy efficiency $\eta$ defined in Supplemenatry Note 1. Here we compare the polydomain BZT films with the main players in the field of capacitive/dielectric energy storage,[1-17] which include ferroelectric polymers and linear polymer dielectrics (used in commercial capacitors), lead-based ferroelectric and anti-ferroelectric films, as well as a few lead-free high energy density ferroelectrics/antiferro-electrics. By comparing the energy storage performance parameters presented in Supplementary Table 1 (a graphic illustration of $W_C$ as function of the applied electric field **E** is given in Supplementary Figure 1), the main achievements of this work can be put into a proper perspective.

In addition to a high energy density, our BZT thin film capacitors are stable over a broad range of temperatures and frequencies. Furthermore, this material has a relatively simple, lead-free composition, which indicates a good manufacturability and environment friendliness. We display in Supplementary Figure 2 the energy and power densities of the engineered polydomain BZT films, together with those of typical electrical devices for energy storage.[18] In this figure, it is clearly shown how this work has changed the landscape of electrical energy storage, i.e., by combining high energy density and high power density into one device. In our future work, we will explore how to reduce the role of a substrate in the energy performance of a ferroelectric film.

**Supplementary Note 3: XRD analysis of the T/R mixed phase structure.**

Supplementary Figure 3a is a representative XRD $2\theta$-scan pattern of the BZT films. Only (*l*00) diffraction peaks are observed for both the bottom electrode layer and the BZT film, with the latter showing a mixture of rhombohedral and tetragonal phases. Supplementary Figure 3b is the $\Phi$-scan pattern of the rhombohedral (101) plane, which shows four strong diffraction peaks with their intervals varying between $89.76^{o}$ and $90.12^{o}$, disclosing a pseudo-cubic heteroepitaxial growth of the BZT film on the electroded substrate.

**Supplementary Note 4: TEM analysis of the BZT films.**

TEM images in Supplementary Figure 4 reveal the typical global and local film morphologies. $1^{st}$ order and $2^{nd}$ order domains of the hierarchical rhombohedral polytwins are shown in Supplementary Figure 4a and Figure 4b, respectively, while Supplementary Figure 4c and Figure 4d in the main text reveal the existence of embedded tetragonal (*T*) domains in the matrix of the rhombohedral (*R*) polytwins.

**Supplementary Note 5: Second Harmonic Generation (SHG) polarimetry analysis.**

To further understand the distribution and evolution of the phase structures in the BZT films, optical second harmonic generation (SHG) polarimetry was performed in the reflection and transmission geometries (Supplementary Figure 5a and Figure 5b). In the reflection geometry, the SHG response arises predominantly from surface domains whereas in the transmission geometry the response is dominated by bulk domains. Supplementary Figure 5c and Figure 5d show *p*- and *s*-polarized reflected SHG intensities as functions of incident light polarization angle from two separate regions of a 1.8 μm thick BZT film. Consistent with the XRD and TEM results, the two regions display tetragonal-dominated (Supplementary Figure 5c) and rhombohedral-dominated phase structures (Supplementary Figure 5d), respectively. This analysis revealed an inhomogeneous distribution of the tetragonal and rhombohedral domains across the surface of the BZT film.

The transmitted SHG responses were also recorded for bulk domain analysis. In this geometry, the *p*- and *s*-polarized SHG intensities (Supplementary Figure 5e and Figure 5f) are attributed to the tetragonal domains oriented along the $[100]$, $[\bar{1}00]$, [010], and $[0\bar{1}0]$ directions, while the *d*-polarized SHG intensity (Fig. 3a and Fig. 3b) is attributed to the rhombohedral domains oriented along the [110], $[\bar{1}10]$, $[\bar{1}\bar{1}0]$, and $[1\bar{1}0]$ directions. Based on analysis of the probed regions, we estimated that the $R$:$T$ phase ratio is about 50:50 for the 0.35 μm thick film, and 62:38 for the 1.8 μm thick film. The rhombohedral domains become more prevalent as the film thickness increases due to relaxation of the strain field.

SHG measurements were carried out at room temperature using a mode-locked Ti:Sapphire ultrashort pulse laser (80 MHz, 10 nJ per pulse, 100 fs) and a regenerative amplifier (250 kHz) as the fundamental light source ($\lambda$=810 nm) with a beam spot of ~ 10 μm in diameter for reflected and transmitted signal detections, respectively. In the reflection and transmission geometries, films were aligned with their surface normal axes at $\theta = 45^o$ and $\theta = 0^o$ with respect to the incident light, respectively. A Glan Polarizer acting as the analyzer and a band pass filter (Thorlabs, FB400-40) were placed in front of a photomultiplier tube module (Hamamatsu, H9305-04) for SHG light polarization selection and filtering out light at the fundamental frequency. A half-wavelength plate was used to rotate the polarizations of the incident laser pulses for collecting *p*-polarized, *s*-polarized, and *d*-polarized SHG signals as functions of the incident light polarization.[19,20]

**Supplementary Note 6: Temperature dependent dielectric performance.**

As shown in Supplementary Figure 6, the outstanding dielectric properties (high $\varepsilon_r$ and low *tgδ*) of the BZT films are stable ( $\varepsilon_r$ reduction ≤10%, *tgδ*≤5%) up to a high temperature of ~300 °C, which leads to the superior energy storage performance at elevated temperatures.

**Supplementary Note 7: Fatigue Test.**

As shown in Supplementary Figure 8, both polarizations and energy densities decreased with cycling times, while the energy efficiency increased with cycling. This indicates that the ratio of the *R* phase (hierarchical polytwins) increased with a cycling electric field.

**Supplementary Note 8: Phase engineering of $BaTiO_3$ via Zr doping.**

In the ferroelectric solid solution of $Ba(Zr_x,Ti_{1-x})O_3$, the room temperature equilibrium phase/bulk phase transforms from a tetragonal phase in pure $BaTiO_3$ into an orthorhombic one and finally a rhombohedral phase with an increasing amount of Zr dopant (Supplementary Figure 9).[21] However, under a biaxial compressive misfit strain, the stable region of the tetragonal phase expands and borders that of the rhombohedral phase.[22] Near room temperature ($T_{\mathrm{rm}}$), these two phases have close free energy densities in epitaxial films (Supplementary Figure 9), and hence a proper misfit will lead to the formation of a tetragonal/ rhombohedral heterophase polydomain structure.

**Supplementary Note 9: Consideration of the misfit strains to create heterophase polydomain BZT films.**

This is done by estimation of the lattice parameters of the metastable tetragonal (*T*) phase. The bulk rhombohedral (*R*) phase of BZT has a lattice constant of 4.06 Å. From the SAED pattern of a BZT film grown on the $LaAlO_3$ substrate, the out-of-plane lattice parameters of the rhombohedral and tetragonal phases (*c*-axis) near the interface are calculated to be 4.21Å and 4.19 Å, respectively. Then an in-plane compressive strain of ~-5.5% can be estimated for the *R* phase at the interface, based on the Poisson's relation (assuming Poisson ratio equals 0.25). This yields an effective substrate lattice parameter at the interface of ~ 3.84 Å, which is a bit larger (extended by the film) than that of the bulk $LaAlO_3$ (3.79 Å). The in-plane strain for the tetragonal phase at the interface was computed to be ~-3.7% by the same method using an estimated bulk tetragonal *c*-lattice parameter of ~4.09 Å (computed from the XRD pattern of the thickest film grown on STO, i.e., 1.8μm BZT on the substrate with the smallest misfit strain, Supplementary Figure 3a). Therefore, based on the same effective substrate lattice parameter (requirement of heteroepitaxy at the interface), the *a*-axis lattice parameter of the *T* phase was estimated: 3.84/(1-0.037)~3.99 Å. The self-strain between a *R* phase and a *T* phase is about (3.99-4.06)/4.06 ~ -1.7%. The choice of the three different substrates with an initial lattice misfit of -3.8% (STO), -4.7% (LSAT) and -6.4% (LAO) was made based on the fact that the films we studied are rather thick (in order to yield a polydomain structure) and buffered by a SRO electrode layer (~-2% misfit with BZT), leading to much smaller misfit strains than the initial ones. This choice was validated by the observations of the *T*/*R* mixed phases in all films on the three substrates with a

thickness of 350nm or thicker, and a single *T* phase in a thin film (~175 nm) grown on the LAO substrate, which has the largest initial compressive misfit with the BZT film. These observations are also consistent with our thermodynamic analysis presented in the main text ("Discussion"), which was schematically shown in Fig. 1d.

**Supplementary References**


1. Chu, B. A Dielectric Polymer with High Electric Energy Density and Fast Discharge Speed. *Science* **313,** 334–336 (2006).
2. Zhou, X., Chu, B., Neese, B., Lin, M. & Zhang, Q. M. Electrical Energy Density and Discharge Characteristics of a Poly(vinylidene fluoride-chlorotrifluoroethylene) Copolymer. *IEEE Trans. Dielectr. Electr. Insul.* **14,** 1133–1138 (2007).
3. Li, Q., Han, K., Gadinski, M. R., Zhang, G. & Wang, Q. High Energy and Power Density Capacitors from Solution-Processed Ternary Ferroelectric Polymer Nanocomposites. *Adv. Mater.* **26,** 6244–6249 (2014).
4. Rabuffi, M. & Picci, G. Status quo and future prospects for metallized polypropylene energy storage capacitors. *IEEE Trans. Plasma Sci.* **30,** 1939–1942 (2002).
5. Khanchaitit, P., Han, K., Gadinski, M. R., Li, Q. & Wang, Q. Ferroelectric polymer networks with high energy density and improved discharged efficiency for dielectric energy storage. *Nat. Commun.* **4,** 2845 (2013).
6. Li, Q. *et al.* Flexible high-temperature dielectric materials from polymer nanocomposites. *Nature* **523,** 576–579 (2015).
7. Kim, Y. *et al.* High-Energy-Density Sol–Gel Thin Film Based on Neat 2-Cyanoethyltrimethoxysilane. *ACS Appl. Mater. Interfaces* **5,** 1544–1547 (2013).
8. Tong, S. *et al.* Lead Lanthanum Zirconate Titanate Ceramic Thin Films for Energy Storage. *ACS Appl. Mater. Interfaces* **5,** 1474–1480 (2013).
9. Liu, Y., Hao, X. & An, S. Significant enhancement of energy-storage performance of $(Pb_{0.91}La_{0.09})(Zr_{0.65}Ti_{0.35})O_3$ relaxor ferroelectric thin films by Mn doping. *J. Appl. Phys.* **114,** 174102 (2013).
10. Zhao, Y., Hao, X. & Zhang, Q. Energy-Storage Properties and Electrocaloric Effect of $Pb_{(1-3x/2)}La_xZr_{0.85}Ti_{0.15}O_3$ Antiferroelectric Thick Films. *ACS Appl. Mater. Interfaces* **6,** 11633–11639 (2014).
11. Hao, X., Zhai, J. & Yao, X. Improved Energy Storage Performance and Fatigue Endurance of Sr-Doped $PbZrO_3$ Antiferroelectric Thin Films. *J. Am. Ceram. Soc.* **92,** 1133–1135 (2009).
12. Ye, M., Sun, Q., Chen, X., Jiang, Z. & Wang, F. Effect of Eu Doping on the Electrical Properties and Energy Storage Performance of $PbZrO_3$ Antiferroelectric Thin

Films. *J. Am. Ceram. Soc.* **94,** 3234–3236 (2011).
13. Wang, Y., Hao, X. & Xu, J. Effects of PbO insert layer on the microstructure and energy storage performance of (042)-preferred PLZT antiferroelectric thick films. *J. Mater. Res.* **27,** 1770–1775 (2012).
14. Do-Kyun Kwon & Min Hyuk Lee. Temperature-stable high-energy-density capacitors using complex perovskite thin films. *IEEE Trans. Ultrason. Ferroelectr. Freq. Control* **59,** 1894–1899 (2012).
15. Love, G. R. Energy Storage in Ceramic Dielectrics. *J. Am. Ceram. Soc.* **73,** 323–328 (1990).
16. Sun, Z. *et al.* Ultrahigh Energy Storage Performance of Lead-Free Oxide Multilayer Film Capacitors via Interface Engineering. *Adv. Mater.* **29,** 1604427 (2017).
17. Park, M. H. *et al.* Thin $Hf_xZr_{1-x}O_2$ Films: A New Lead-Free System for Electrostatic Supercapacitors with Large Energy Storage Density and Robust Thermal Stability. *Adv. Energy Mater.* **4,** 1400610 (2014).
18. Whittingham, M. S. Materials Challenges Facing Electrical Energy Storage. *MRS Bull.* **33,** 411–419 (2008).
19. Kumar, A., Prakash, J., Khan, M. T., Dhawan, S. K. & Biradar, A. M. Memory effect in cadmium telluride quantum dots doped ferroelectric liquid crystals. *Appl. Phys. Lett.* **97,** 163113 (2010).
20. Mishina, E. D. *et al.* Domain orientation in ultrathin $(Ba,Sr)TiO_3$ films measured by optical second harmonic generation. *J. Appl. Phys.* **93,** 6216–6222 (2003).
21. Yu, Z., Ang, C., Guo, R. & Bhalla, A. S. Piezoelectric and strain properties of $Ba(Ti_{1-x}Zr_x)O_3$ ceramics. *J. Appl. Phys.* **92,** 1489–1493 (2002).
22. Zeches, R. J. *et al.* A Strain-Driven Morphotropic Phase Boundary in $BiFeO_3$. *Science* **326,** 977–980 (2009).
